# Frugivorous bats in the Colombian Caribbean region are reservoirs of the rabies virus

**DOI:** 10.1186/s12941-019-0308-y

**Published:** 2019-03-19

**Authors:** Alfonso Calderón, Camilo Guzmán, Salim Mattar, Virginia Rodríguez, Arles Acosta, Caty Martínez

**Affiliations:** 1 Instituto de Investigaciones Biológicas del Trópico (IIBT), Facultad de Medicina Veterinaria y Zootecnia, Doctorado en Medicina Tropical, Universidad de Córdoba, Carrera 6 No 76-103., Monteria, Córdoba Colombia; 2 Instituto de Investigaciones Biológicas del Trópico (IIBT), Programa Regencia en Farmacia, Facultad de Ciencias de la Salud, Doctorado en Medicina Tropical, Universidad de Córdoba, Monteria, Colombia; 3 Grupo de Investigaciones Microbiológicas y Biomédicas de Córdoba (GIMBIC). Programa de Bacteriología, Facultad de Ciencias de la Salud, Universidad de Córdoba, Monteria, Colombia; 4 Producción Animal Tropical Facultad de Medicina Veterinaria y Zootecnia, Universidad de Córdoba, Monteria, Colombia

**Keywords:** Chiroptera, Ecosystems, Epidemiology, Public health, Transmission, Zoonoses

## Abstract

**Background:**

Bats are an important ecological group within ecosystems. The rabies virus is a *Lyssavirus*, and haematophagous bats are the principal reservoir; however, the virus has also been detected in non-haematophagous bats. The objective was to determine the rabies virus in non-haematophagous bats in the Colombian Caribbean region.

**Methods:**

In 2017, a cross-sectional study was carried out with a base-risk sampling in twelve geographic zones of the Colombian Caribbean area that included the main ecosystems of two departments. 286 bats were captured, which were euthanized with a pharmacological treatment following the ethical protocols of animal experimentation. The taxonomic identification was done with dichotomous keys. The necropsy was carried out at the capture site, and brain samples were kept in liquid nitrogen. The extraction of the RNA was carried out from the frozen brains with Trizol™; a fragment of 914 bp of the glycoprotein G of the rabies virus was amplified with RT-PCR. The amplicons were sequenced with the Sanger method.

**Results:**

Twenty-three genera of bats were identified, and, in two frugivorous, *Artibeus lituratus* and *Artibeus planirostris,* amplicons were obtained and sequenced as the rabies virus.

**Conclusions:**

This is the first evidence of natural infection of the rabies virus in frugivorous bats in the Colombian Caribbean area; this result is important for the surveillance and control of rabies.

## Background

Rabies is a zoonotic disease that affects humans through saliva, bites or scratches [1]. The natural hosts of the rabies virus include Carnivora and Chiroptera [2]. The rabies virus belongs to the genus *Lyssavirus* and produces fatal acute encephalitis in humans [3]. Rabies is distributed on all continents, except Antarctica [3]; once the symptoms appear, the disease is fatal [1]. Global mortality is estimated at 59.000 human cases per year, 95% of these cases occur in Africa and Asia, mainly from dog bites [4]. In developed countries, wild species are the principal reservoirs, and, in domestic animals, mass vaccination prevents the spread of the virus [5].

Bats are an important ecological group in nature because of their ability to control insects, disperse seeds [6] and pollinate [7]. 70% of bat species are insectivorous and widely distributed worldwide [8]. Bats are hosts of high viral diversity, with a high zoonotic potential [9]. The rabies virus and other *Lyssavirus* do not appear to cause disease in bats, suggesting co-evolution between the viruses and their hosts [10, 11]; high colony densities of up to 3000 bats per square meter [12] and repeated infections are likely to occur frequently, providing a mechanism for resistance to rabies [13]. Bats can carry a large number of infectious agents, but they do not suffer from the disease, and it is believed that the increase in body temperature as a result of flight increases the metabolic rate, activating mitochondria to trigger the immune cascade with interleukin production and prostaglandins, avoiding infection with the pathogens that they carry [14].

The haematophagous bats *Desmodus rotundus, Diaemus youngi* and *Diphylla ecaudata* are reservoirs of the rabies virus and are distributed from the Tropic of Cancer to the Tropic of Capricorn [15]. These species of haematophagous bats are involved in the transmission of rabies in the tropics. In Colombia, like other countries in the Caribbean, Central America and South America, the majority of cases of human rabies transmitted by haematophagous bats have been associated with *D. rotundus* [16].

There are two cases reported of human rabies apparently transmitted by non-hematophagous bats. The first was reported in the USA in 1953 [17] and the second one was in 1996 in Chile [18]. The serological and genetic characterization denoted that the reservoir in Chile was the insectivorous bat *Tadarida brasiliensis* [18]. In the Americas, there are several species of insectivorous, frugivorous, nectarivorous, omnivorous and carnivorous bats that have been reported as reservoirs for the rabies virus [19].

On the other hand, according to the Colombian Agriculture Institute (ICA), there have been 184 cases of encephalitis [20]; the National Institute of Health of Colombia (INS) reported 13 human cases compatible with encephalitis up to week 38 of 2018 [21]. There are several problems one of them is the sub-registrations report of animal cases that don’t allow a full epidemiological analysis. Also the recollection methods of the sample are very deficient, resulting in a poor diagnosis at the laboratory level. On the other hand, recently in Colombia in some areas of the Departments of Cordoba and Sucre, bat bites were observed in cows and horses, and hematophagous bats were captured, in which the rabies virus was detected in the brains with a prevalence of 2% with Sellers staining technique and 13% with direct immunofluorescence [22, 23]. The sensitivity and specificity of immunohistochemistry (IHC) and direct immunofluorescence (DIF) depending on the clinical status of the pathology at the moment of the diagnosis; the sensitivity and specificity of ICH is 70% when Negri bodies are present and the DIF is of 100% [24]. Regarding the occurrence of the rabies virus in non-hematophagous bats, there have only been two previous studies in Colombia one in 1968 in San Vicente de Chucurí (Santander) in the low tropic of Magdalena River, a rabies virus was found in *Carollia perspicillata* [25]. The second one was reported in 2012 in the Western area of the country (Cali) in urban bats Molossus *molossu*s and *Eptesicus brasilensis* [16]. In Cordoba and Sucre departments, there are no ecoepidemiological studies on the circulation of the rabies virus in non-hematophagous bats. The objective of this study was to detect the rabies virus in non-haematophagous bats in two departments of the Colombian Caribbean region.

## Methods

### Type of study, study area and sampling

**Fig. 1:**
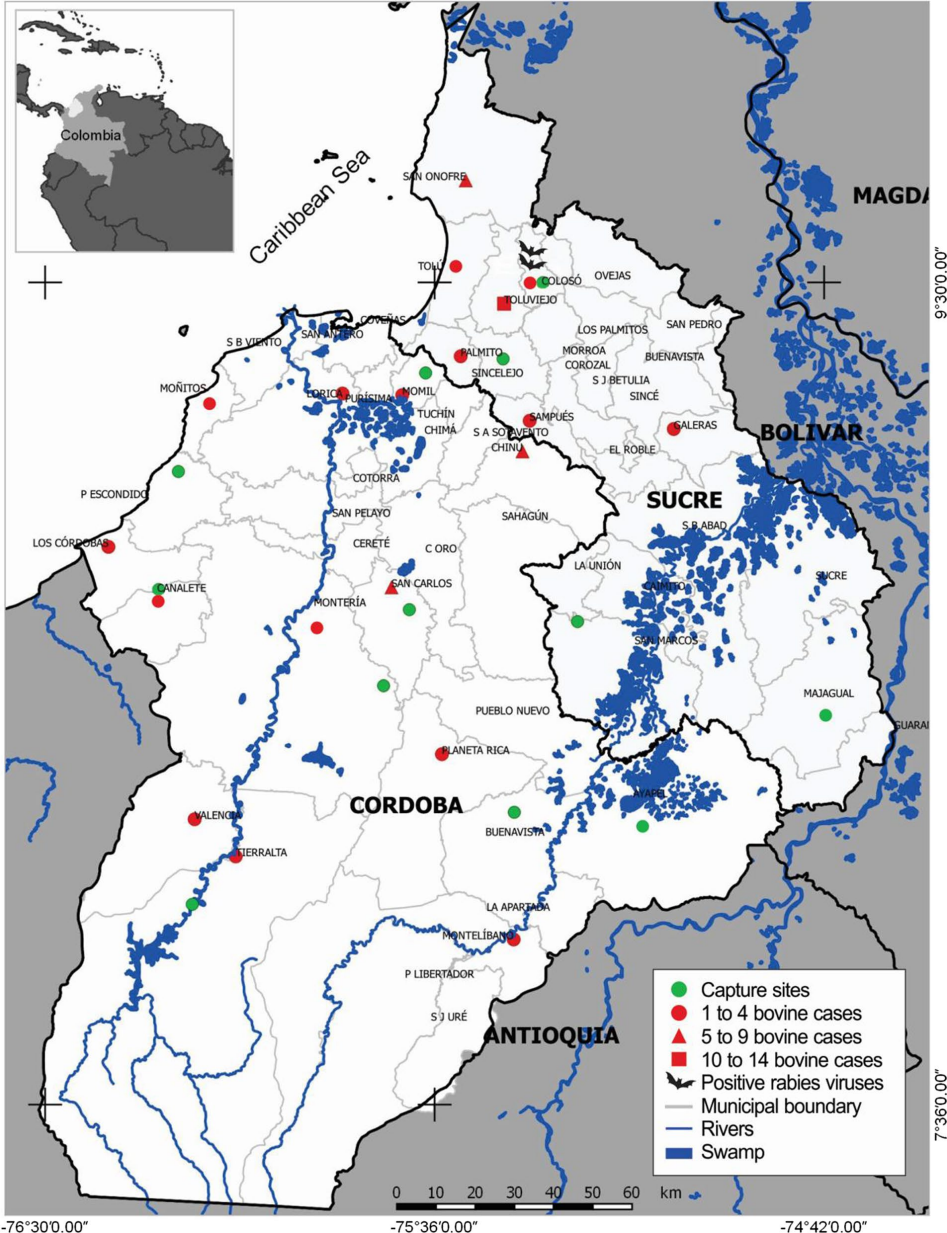
Distribution of cases of bovine rabies in Cordoba and Sucre reported by ICA during 2014–2016 and sampling sites used in the present study

In 2017, in two departments of the Colombian Caribbean region, a cross-sectional study was carried out with a base-risk sampling, for which 286 bats were captured. Based on the fact that, during 2014 and 2016 in the departments of Cordoba and Sucre, there were 146 cases of bovine rabies, according to the Colombian Agriculture Institute [26], 12 sampling sites were selected in these endemic areas for the rabies virus (Fig. 1). Of the 12 sampling sites, 8 were in Cordoba and 4 in Sucre; in both departments, the main ecosystems were included (Fig. 1).

### Capture of specimens

The bats were captured using five mist nets for 4 h of sampling, which corresponded to 240 h/network. The bats were identified with dichotomous taxonomic keys based on morphometric parameters [27]. The bats were initially medicated with atropine (0.005 mg/kg, Laboratories ZOO™, Colombia) and acepromazine (0.11 mg/Kg; Laboratories ZOO™, Colombia) using intramuscular administration and euthanized with an intracardiac overdose of 0.2 ml of sodium pentobarbital (Invet™, Colombia). The dissection was performed at the capture site where the brains were removed and deposited in sterile cryovials with Trizol™ (Invitrogen™) and kept in tanks with liquid nitrogen. To preserve the species, pregnant or lactating females were released.

### Molecular detection of the rabies virus

The RNA extraction was performed with Trizol™ (Invitrogen) from 286 samples of brain tissues; the aliquots were resuspended in 150 μL of nuclease-free water. The concentration of the RNA obtained with the NanoDrop 2000 equipment (Spectrophotometers™) was then quantified. The cDNA synthesis was done with the reverse transcriptase enzyme M-MLV (Invitrogen™) using random primers (Invitrogen™), following the recommendations of the manufacturers. Subsequently, a conventional PCR was carried out that amplified a fragment of the gen G with the primers (Ga3222-4) (5′CGCTGCATTTTRTCARAGT3′) and (Gb4119-39) (5′GGAGGGCACCATTATTGGTMTC3′), which amplify a fragment of 914 bp [3, 28]. The initial denaturation was done at 94 °C for 5 min, then 35 cycles at 94 °C for 45 s, 55 °C for 45 s and 72 °C for 90 s and a final extension at 72 °C for 5 min. As a control of species and internal control, complementary primers of a sequence of the mitochondrial gen mt DNA of bats were used [29]. As a positive control, RNA extracted with Trizol™ (Invitrogen) was used from the vaccine (strain PM/WI38, Lyon, France), and molecular biology grade water was used as a negative control. Agarose-gel electrophoresis was carried out with the amplification products. The amplicons were sequenced with the Sanger method was carried in Korea (Macrogen™).

### Phylogenetic analyzes

The sequences were edited and aligned automatically with Geneious software (version 9.1.79), where two sequences of 793 bp were obtained. These sequences were aligned with Clustal W, found in Mega7. For the phylogenetic reconstructions, 86 reference sequences available in the GenBank were used for typing by groups. The sequence of the vaccine strain was also included. The best evolution model was applied to explain the nucleotide diversity observed between the aligned sequences, and the phylogenetic reconstructions were done using Neighbor-Joining (NJ), Maximum Likelihood (ML) and Unweighted Pair Group Method with Arithmetic Mean (UPGMA) using the software MEGA7.

## Results

**Fig. 2:**
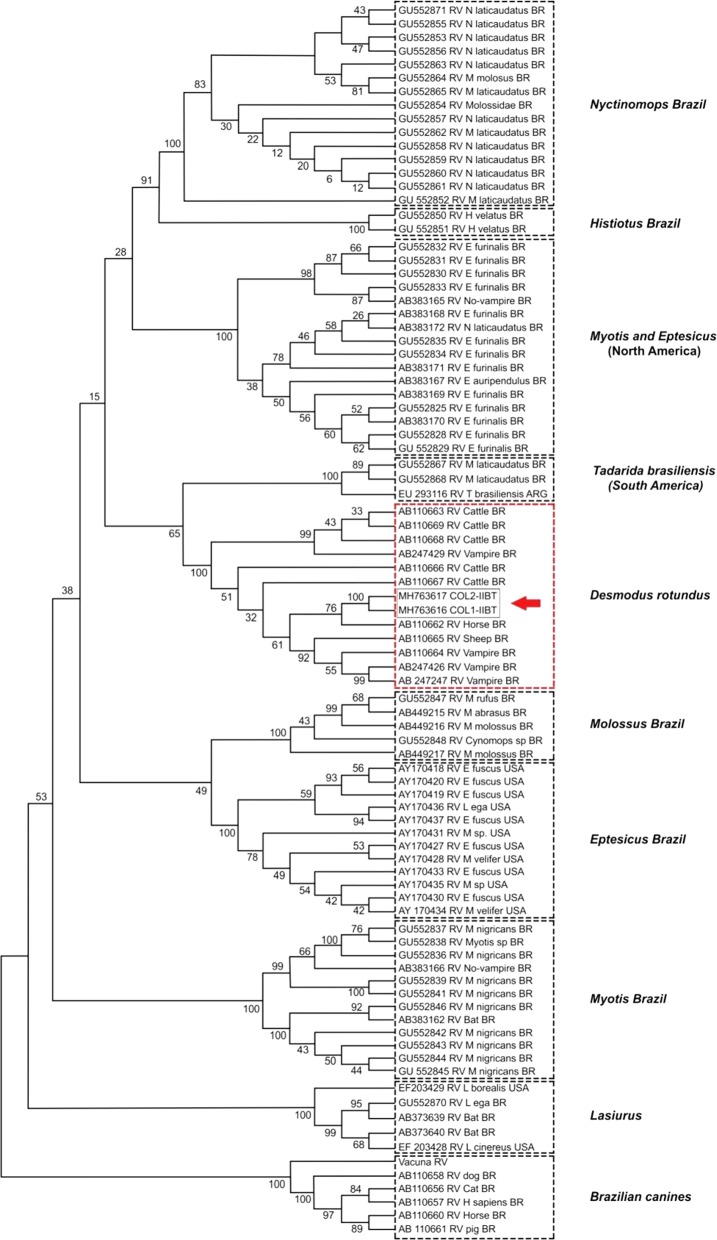
Phylogenetic reconstruction with Maximum Likelihood for gen G of *Lyssaviru*s; in the red box are the two sequences detected in the frugivorous bats of the present study in Coloso (Sucre)

In the twelve sampling sites, 286 bats were captured, distributed in six families and 23 species. Table 1 shows the distribution of the bats and their food habits. Amplicons of the glycoprotein G of the rabies virus were detected in two brains of frugivorous bats *A. lituratus* and *A. planirostris,* captured in Coloso, Department of Sucre (latitude 9.4980^o^N, longitude − 75.3494^o^W). Four *D. rotundus* specimens were captured in this study, two in San Carlos, Cordoba (latitude 8.7441^o^N, longitude − 75.6563^o^W) and two in Coloso (Sucre), both were negative for the rabies virus with the RT-PCR. The sequences detected in the frugivorous bats were recorded in the GenBank under the numbers MH763616 and MH763617. These two sequences were grouped within the clade *D. rotundus* or rabies virus of sylvatic origin (Fig. 2), with an average internal distance between the taxa of 3%.

**Table 1:** Distribution of chiroptera species and food habits in the sampling sites of Cordoba and Sucre

Species	Sample sites												
	Cordoba								Sucre				
	Ayapel	Buena vista	Canalete	Momil	Monteria	P. Escondido	San Carlos	Tierralta	Coloso	Majagual	San Marcos	Sincelejo	Total
Frugivorous													
*Artibeus planirostris*	6	14	4	18	13	8	8		9	7	3	9	99
*Carollia perspicillata*	5	3			6	1	2	17	1	2		1	38
*Artibeus lituratus*	2	1	1	3	3	10	3		1	1	4	1	30
*Sturnira lilium*	1	1	3			1	1	4				9	20
*Carollia brevicauda*								1					1
*Carollia castanea*												1	1
*Urodema bilobatum*	1	1		2	2	1					4		11
Insectivorous													
*Molossus molossus*	5									5	4		14
*Sacopterix bilineata*								2	1		1		4
*Eptesicus brasilensis*										1			1
*Rhogeessa io*			1				1						2
*Eumops glaucinus*	1												1
*Lasiurus ega*				1									1
*Micronycteris microtis*							1						1
*Myotis nigricans*		1											1
*Sacopterix leptura*										1			1
*Molossops temminckii*								1					1
Nectarivorous													
*Glossophaga soricina*			1	1		1			1		1	1	6
Haematophagous													
*Desmodus rotundus*							2		2				4
Piscivorous													
*Noctilio albiventris*	1	1			1								3
*Noctilio leporinus*											3		3
Omnivorous													
*Phyllostomus discolor*	3	2	13			1	5		10	5	2	1	42
*Trachops cirrhosus*												1	1
Total	25	24	23	25	25	23	23	25	25	22	22	24	286

The numbers represent the bats captured in each geographical sites

## Discussion

The rabies virus was detected in two brains of frugivorous bats, *A. lituratus* and *A. planirostris*. These findings are consistent with a report for *A. lituratus* in Bolivia and *A. planirostris* in Argentina, Belize, Bolivia, Brazil, Guatemala, Mexico, Peru and Trinidad and Tobago, where in non-haematophagous bats have detected the rabies virus [19].

In the urban area of Capanema (Brazil), a high percentage of seropositivity (52.46%) was detected in *A. planirostris* to the rabies virus, but the brain was not positive for the infection. This high seropositivity indicated that the rabies virus can be spread in urban areas [30]. Studies on insectivorous bats have shown protective neutralizing antibodies for several years; however, these antibodies would not prevent bats from becoming sick and dying of rabies [31]. It has also been proposed that large population sizes and overcrowded conditions in roosts facilitate intra- and interspecies transmission [32].

On the other hand, the phylogenetic analysis showed 304 variable sites of 793 analyzed sites, and a total of 203 parsimoniously informative sites were determined. The phylogenetic analyzes showed ten clades with an average distance of 14.3%. The sequences MH763616 and MH763617 were grouped with 100% branch support within the sequences that are directly related to the rabies virus isolated from *D. rotundus*; this bat is the principal vector, with high viremia and the ability to infect other animals. *D. rotundus* uses different animal species to obtain the blood, or by with high grooming social interactions [33, 34] or by the sympatry when they share shelters [35]. The topology of the phylogenetic tree shows ten clades grouped by different hosts and the sequences of the viruses detected in them and coincide with that of Oliveira [3], who reported ten clades similar to the present study.

In non-hematophagous bats from Sao Paulo (Brazil), three antigenic variants were identified (AgV-3, AgV-4 and AgV-6), all previously identified in Latin America [36–38]. These variants represent reservoirs of the Latin American rabies virus maintained in populations of the bats *Desmodus rotundus* (AgV-3), *Tadarida brasiliensis* (AgV-4) and *Lasiurus cinereus* (AgV-6) [39]. In Colombia, three genetic variants were identified of urban rabies cases that involved dogs, humans and non-haematophagous bats. The Colombian variant I was found in the Andean region and in the Department of Arauca (close to Venezuelan border); in this department, the last case of rabies was detected in a dog in 1977, and mass vaccination is believed to have eliminated variant I. Variant II was detected in the Colombian Caribbean, and two cases of haematophagus bats were found, one in a human and another in a dog. Variant III was found in southern Colombia in the Department of Valle del Cauca in two insectivorous bats (*M. molossus, E. brasiliensis*), in three dogs and one human [40]. The sequences found in the present study (MH763616.1 and MH763617) possibly are related to group I to the vampire bat *D.* *rotundus* (AgV3). The circulation of the genetic variants of the rabies virus in bats and other mammals is important because it establishes the virus and determines the risks to public health. The rabies virus variants are a significant public health concern; thus, the entire rabies viruses are potentially infectious for human’s beings. Previous studies in Colombia showed that rabies virus transmitted in epizootic outbreaks, the viruses were closely genetic related. Hence, reservoirs can carry any infectious of rabies variant [40, 41]. The present study cannot explain how it is the Colombian eco-epidemiological situation of rabies in different geographical areas like the Caribbean, Savannas (Llanos Orientales) and Pacific area for example, situation that it was explained by Hutter et al. in Costa Rica, who found association between animal rabies and rainy season [42].

The control of wild-origin zoonoses is limited by the insufficient knowledge on the biology of pathogens in the hosts, which coincides with pulses of viral excretion within bat populations produce densities changes and therefore contact rates [43]. Viruses can disappear locally, but persist globally through migration and new outbreaks in subsequent generations or decreased immunity, allowing circulation of the virus within the group or circulation of persistently infected bats. These concepts are applicable to a wide range of pathogens that affect humans and domestic and wild animals [43, 44].

## Conclusions

This study presents the first molecular evidence of the natural infection of the rabies virus in frugivorous bats, *A. lituratus* and *A. planirostris,* captured in the Colombian Caribbean region. It is necessary to expand to other areas of the Colombian Caribbean and compare with other regions like the Pacific area, to observe better the biology and behavior of these animals and their involvement in the transmission of rabies.

## Abbreviations

WHO: World Health Organization
ICA: Colombian Agriculture Institute
INS: National Institute of Health (Colombia)
NJ: neighbor-joining
ML: maximum likelihood (ML)
UPGMA: unweighted pair group method with arithmetic mean
MEGA: molecular evolutionary genetics analysis
AgV-3: antigenic variants 3
AgV-4: antigenic variants 4
AgV-6: antigenic variants 6
